# Deep Learning-Based Stage-Wise Risk Stratification for Early Lung Adenocarcinoma in CT Images: A Multi-Center Study

**DOI:** 10.3390/cancers13133300

**Published:** 2021-06-30

**Authors:** Jing Gong, Jiyu Liu, Haiming Li, Hui Zhu, Tingting Wang, Tingdan Hu, Menglei Li, Xianwu Xia, Xianfang Hu, Weijun Peng, Shengping Wang, Tong Tong, Yajia Gu

**Affiliations:** 1 Department of Radiology, Fudan University Shanghai Cancer Center, 270 Dongan Road, Shanghai 200032, China; jinggong@shca.org.cn (J.G.); haimingli001@gmail.com (H.L.); zhuhui573@gmail.com (H.Z.); wtingting714@gmail.com (T.W.); h16211230001@gmail.com (T.H.); leim11898@gmail.com (M.L.); wjunp2020@gmail.com (W.P.); 2 Department of Oncology, Shanghai Medical College, Fudan University, Shanghai 200032, China; 3 Department of Radiology, Shanghai Pulmonary Hospital, 507 Zheng Min Road, Shanghai 200433, China; jiyuliu001@gmail.com; 4 Department of Radiology, Municipal Hospital Affiliated to Taizhou University, Taizhou 318000, China; xianwuxia001@gmail.com; 5 Department of Radiology, Huzhou Central Hospital Affiliated Central Hospital of Huzhou University, 1558 Sanhuan North Road, Huzhou 313000, China; hzszxyy@hzhospital.com

**Keywords:** risk stratification, ground glass nodule, lung adenocarcinoma, deep learning, CT image

## Abstract

**Simple Summary:**

Prediction of the malignancy and invasiveness of ground glass nodules (GGNs) from computed tomography images is a crucial task for radiologists in risk stratification of early-stage lung adenocarcinoma. In order to solve this challenge, a two-stage deep neural network (DNN) was developed based on the images collected from four centers. A multi-reader multi-case observer study was conducted to evaluate the model capability. The performance of our model was comparable or even more accurate than that of senior radiologists, with average area under the curve values of 0.76 and 0.95 for two tasks, respectively. Findings suggest (1) a positive trend between the diagnostic performance and radiologist’s experience, (2) DNN yielded equivalent or even higher performance in comparison with senior radiologists, and (3) low image resolution reduced the model performance in predicting the risks of GGNs.

**Abstract:**

This study aims to develop a deep neural network (DNN)-based two-stage risk stratification model for early lung adenocarcinomas in CT images, and investigate the performance compared with practicing radiologists. A total of 2393 GGNs were retrospectively collected from 2105 patients in four centers. All the pathologic results of GGNs were obtained from surgically resected specimens. A two-stage deep neural network was developed based on the 3D residual network and atrous convolution module to diagnose benign and malignant GGNs (Task1) and classify between invasive adenocarcinoma (IA) and non-IA for these malignant GGNs (Task2). A multi-reader multi-case observer study with six board-certified radiologists’ (average experience 11 years, range 2–28 years) participation was conducted to evaluate the model capability. DNN yielded area under the receiver operating characteristic curve (AUC) values of 0.76 ± 0.03 (95% confidence interval (CI): (0.69, 0.82)) and 0.96 ± 0.02 (95% CI: (0.92, 0.98)) for Task1 and Task2, which were equivalent to or higher than radiologists in the senior group with average AUC values of 0.76 and 0.95, respectively (*p* > 0.05). With the CT image slice thickness increasing from 1.15 mm ± 0.36 to 1.73 mm ± 0.64, DNN performance decreased 0.08 and 0.22 for the two tasks. The results demonstrated (1) a positive trend between the diagnostic performance and radiologist’s experience, (2) the DNN yielded equivalent or even higher performance in comparison with senior radiologists, and (3) low image resolution decreased model performance in predicting the risks of GGNs. Once tested prospectively in clinical practice, the DNN could have the potential to assist doctors in precision diagnosis and treatment of early lung adenocarcinoma.

## 1. Introduction

Lung cancer is the leading cause of cancer-related deaths globally, with almost one-quarter of all cancer deaths [1]. The popularization of low-dose computed tomography (CT) screening reduced the mortality of lung cancer significantly [2]. Early lung cancer screening through detection and diagnosis of pulmonary nodules on CT scans is an essential and effective method. A large fraction of ground glass nodules (GGNs) are detected on the screening of CT images. As the biopsy of GGNs is a difficult task for interventional physicians, CT imaging is one of the optimal diagnosis measures for GGNs, especially for small ones. Most malignant GGNs are histopathologically confirmed as early-stage lung adenocarcinomas. According to the classification of the International Association for the Study of Lung Cancer/American Thoracic Society/European Respiratory Society, early-stage lung adenocarcinomas consist of pre-invasive lesions involving atypical adenomatous hyperplasia and adenocarcinoma in situ (AIS), minimally invasive adenocarcinoma (MIA), and invasive adenocarcinoma (IA) [3]. The 5-year disease-free survival rates of patients diagnosed with AIS and MIA are close to 100%, which are higher than that of IA patients (40%–85%) [4]. Therefore, a precise diagnosis of GGNs facilitates the classification of low- and high-risk individuals (i.e., patients with benign and malignant GGNs, respectively), thereby avoiding overdiagnosis or overtreatment for early lung adenocarcinoma [5]. It is also possible to make a personalized clinical care plan and select the optimal surgical treatment for patients with different pathological types (i.e., IA and non-IA patients).

To diagnose and discriminate the subtypes of lung adenocarcinoma, some studies proposed and developed a quantitative imaging method to quantify the image features of GGNs for discrimination model development [6,7]. The quantitative imaging features can depict the properties of GGNs in shape, CT value distribution, and texture aspects [8]. To improve the performance, the radiomics model was developed to extract thousands of image features to decode the CT imaging phenotypes of GGNs [9,10,11]. The radiomics model consists of tumor segmentation, feature extraction and selection, classifier training/testing, and performance evaluation processes [12]. The CT-based radiomics feature reflects the internal heterogeneity of GGNs well.

Meanwhile, an end-to-end convolutional neural network (CNN) was applied to build deep neural network (DNN) models to classify the subtypes of GGNs [13,14]. The DNN model derives high dimensional hierarchy imaging features from the internal and surrounding regions of GGNs on CT images without tumor segmentation and handcrafted feature extraction [15,16,17]. Machine learning and DNNs have been successful in predicting tumor molecular features, treatment response, and prognosis in the oncology of lung cancer. Previous studies have developed computer-aided detection/diagnosis (CADe/CADx) models to detect nodules on CT images and evaluate the histopathologic type of GGNs by using DNNs [16,18]. Compared with the CT-based radiomics model, a DNN-based model improves the detection and classification performance significantly.

To develop a highly efficient DNN-based CAD model, several studies have employed state-of-the-art deep learning architectures in the computer vision domain to extract tumor features directly from CT images and generate features adaptive to a given lung cancer risk stratification problem. Among these DNN architectures, ResNet and DenseNet are the most popular in lung cancer diagnosis [18,19]. Since there is a lack of large and general enough datasets, a number of studies used a transfer learning technique to build DNN models. Several studies applied a multi-task learning strategy to reduce overfitting for limited datasets, e.g., combining classification and segmentation tasks to develop multi-task DNN models. Moreover, based on the dimensions of input images, the DNNs can be categorized as 2D, 2.5D, and 3D. As anatomical structures of GGNs appear as a 3D shape on CT scans, the development and application of a 3D DNN may be an optimal way to predict the risk of early-stage lung adenocarcinoma.

Hence, this study developed a two-stage DNN model to diagnose benign and malignant GGNs (Task1) and classify between IA and non-IA tumors (Task2) by using a 3D convolutional neural network. Then, the histopathologically confirmed GGNs collected from four centers were used to train and test the DNN. Finally, a multi-reader multi-case (MRMC) observer study was conducted to evaluate the model performance by comparing the performance of six radiologists and the DNN in early lung adenocarcinoma risk stratification.

### 1.1. Related Works

To predict the risk of early-stage lung adenocarcinoma, researchers have developed various CADx models by using CT images. Gao et al. [20] analyzed CT findings (i.e., lesion boundary, average CT value, etc.) of GGNs to develop classification models. Although the application of the CT signs is feasible to classify different pathology types of GGNs, the evaluation features rely heavily on the radiologist’s subjective interpretation.

Quantitative CT imaging or radiomics feature analysis has been developed to quantify the image features of GGNs because of its ability to decode imaging phenotypes of the intra-tumor heterogeneity [12,21,22,23]. Zhao et al. [24] developed a radiomics-based nomogram, which incorporates both a radiomics signature and mean CT value, for differentiation of pre-invasive lesions from invasive lesions that appear as GGNs. Although the human-engineered radiomics features are effective to predict the invasiveness of GGNs with small datasets, radiomics model development is time consuming and human labor intensive (e.g., tumor segmentation) which limits its repeatability and application. Still, tumor segmentation and feature extraction processes need the radiologist’s subjective intervention (i.e., GGN boundary delineation) and a pre-defined handcrafted image feature extractor, which may cause subjective biases.

DNN is another promising tool for early-stage lung adenocarcinoma risk stratification [16]. Wang et al. [25] proposed a 3D CNN-based classification framework consisting of nodule detection and cancer classification to diagnose pre-invasive and invasive GGNs. Gong et al. [18] developed a residual learning-based CNN model to classify between IA and non-IA GGNs, which improved the classification performance. Wang et al. [26] proposed a multi-task deep learning model with both segmentation and classification networks, which showed that the segmentation can better facilitate the classification of pulmonary GGNs. Overall, as an end-to-end architecture, the DNN model not only achieves higher prediction accuracy compared with a radiomics model, but also saves human labor in delineating the GGN boundary. Thus, it is more applicable and repeatable than a radiomics model.

Recently, the combined radiomics and deep learning models were investigated to develop a multiple feature fusion model for GGN classification. Wang et al. [27] proposed a combined deep learning and radiomics classification model to classify IA from non-IA, which showed higher performance in comparison with a single feature-based model. Hu et al. [28] compared and integrated the deep learning and radiomics features to develop a CADx model to classify benign and malignant, which also demonstrated that a fusion model can improve classification performance. Although fusion of deep learning and radiomics features is feasible to improve the model performance, extracting radiomics features also needs a large amount of labor and its application is more difficult due to the complex model design.

All the aforementioned studies are either involved classifying between benign and malignant GGN or predicting invasiveness of IA. As a multi-phase task, the lung adenocarcinoma risk can be more comprehensively predicted by using a stage-wise risk stratification model. Thus, a two-stage DNN model is developed to predict malignancy and invasiveness of GGNs due to the high accuracy and good repeatability of deep learning. Since achieving a highly accurate DNN model requires a large amount of training data, we collected GGNs from four centers to build a robust deep learning model. Moreover, conducting an observer study can better compare and evaluate the performance of the DNN and radiologists with different levels of experience. An MRMC observer study with six radiologists’ participation was conducted. To our knowledge, there has not yet been a two-stage DNN model to stratify the risk of lung adenocarcinoma with large multi-center datasets and an MRMC study.

### 1.2. Contributions

Our contributions can be summarized as follows: (1) In this study, we proposed and developed a DNN model to stratify the risk of early lung adenocarcinoma by using CT images. The two-stage model not only classified between benign and malignant GGNs, but also predicted the invasiveness of malignant tumors by differing IA from non-IA. (2) By conducting an MRMC observer study, our result demonstrates that the deep learning model performed equivalent to or even better than senior radiologists in predicting the risk of GGNs. (3) Analyzing the DNN performance changes on CT images with different resolutions, we found that the low resolution of CT images decreased the model performance.

The rest of the paper is organized as follows. Section 2 introduces the detail of the dataset and proposed two-stage DNN model. Section 3 presents the experimental results. Section 4 discusses the characteristics and limitations of this study. Section 5 concludes the paper.

## 2. Materials and Methods

### 2.1. Datasets

A total of 2393 GGNs collected from 2105 patients in four centers were used to develop the DNN model. There were 1476, 431, 284, and 202 GGNs in the training dataset (center 1: Fudan University Shanghai Cancer Center), tuning dataset (center 2: Huzhou Central Hospital), validation dataset 1 (center 3: Taizhou Municipal Hospital), and validation dataset 2 (center 4: Shanghai Pulmonary Hospital), respectively. Table 1 summarizes and lists the characteristics of patients in the four datasets. The inclusion criteria were: (1) GGN with a diameter in the range [3 mm, 30 mm] on the chest CT image, (2) the surgically histopathologically confirmed tumor was benign or stage I lung adenocarcinoma (involving AIS, MIA, and IA), (3) available CT examination within one month before surgery, and (4) available CT image in digital imaging and communications in medicine format. The exclusion criteria were: (1) lack of CT scan, (2) history of neoadjuvant systemic therapy or other therapy, (3) histopathologically confirmed GGN was not identifiable on CT image, and (4) history of cancer before surgery. The details of the CT scanner manufacturer and convolutional kernel are shown in Appendix B Table A1. Each GGN was treated as an independent primary lesion, as well as the case with multi-focal GGNs. The center position of each GGN was marked by reviewing the histopathology report and CT scans obtained before and after surgery. The X, Y, and Z coordinates of the center point in the 3D image matrix were recorded to locate the position of GGNs on the CT scan.

The institutional review boards (IRBs) in four centers approved this multi-center study, and the requirements for informed consent forms were waived due to its retrospective nature. This study was conducted in accordance with the Declaration of Helsinki and approved by the IRB of Fudan University Shanghai Cancer Center (protocol code: 2103232-24), Huzhou Central Hospital (protocol code: 20180738-01), Taizhou Municipal Hospital (protocol code: LW013), and Shanghai Pulmonary Hospital (protocol code: K18-204Y).

### 2.2. Two-Stage DNN Model Development

#### 2.2.1. Image Pre-Processing

**Figure 1:**
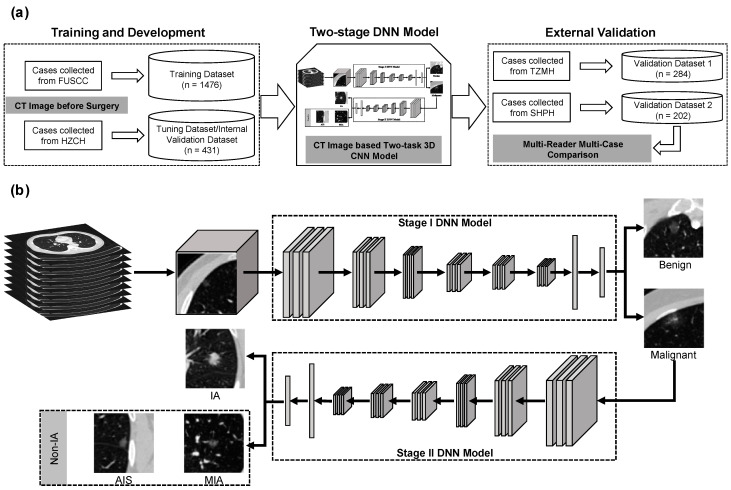
The workflow of model development. (**a**) Schematic workflow of the study for training and external validation of a CT image-based DNN model. (**b**) Flowchart of the proposed two-stage DNN model. The stage I DNN model was used to classify benign and malignant GGNs. The stage II DNN model was used to predict the invasiveness risk of malignant tumors. FUSCC = Fudan University Shanghai Cancer Center. HZCH = Huzhou Central Hospital. TZMH = Taizhou Municipal Hospital. SHPH = Shanghai Pulmonary Hospital.

**Figure A1:**
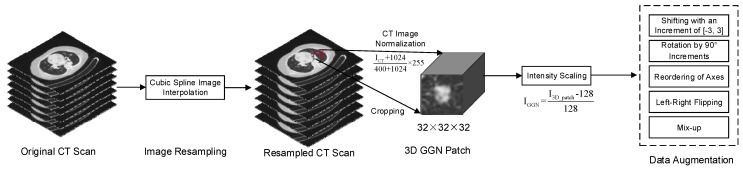
The flowchart of image pre-processing step.

A two-stage DNN model was developed to build the risk stratification scheme of GGNs. Figure 1 shows the flowchart of the proposed two-stage DNN model. To build the two-stage DNN model, the 3D CT image was firstly resampled with a voxel size of 1 mm × 1 mm × 1 mm by using a cubic spline image interpolation algorithm. Then, the CT values of each scan were normalized to [0, 255] by applying a window range of [−1024HU, 400HU]. A 32 mm × 32 mm × 32 mm cubic of each GGN was cropped from a normalized 3D image based on the coordinate values of the center point. The gray value of each cropped 3D patch was transformed into [−1, 1] by using a scale mapping of $\frac{I_{3D\_patch}-128}{128}$. Appendix B Figure A1 illustrates the flowchart of the image pre-processing step.

#### 2.2.2. Data Augmentation

A series of data augmentation techniques were applied to increase the number of samples in the training dataset. These techniques were as follows: (1) shifting the center point of GGNs with an increment of [−3, 3] voxels in each axis, (2) rotation of the 3D patch by 90° increments in three axes, (3) reordering the axes, (4) left–right flipping. To improve the performance of the DNN, the data augmentation process was performed on the fly during the model training process.

#### 2.2.3. DNN Model

**Figure A2:**
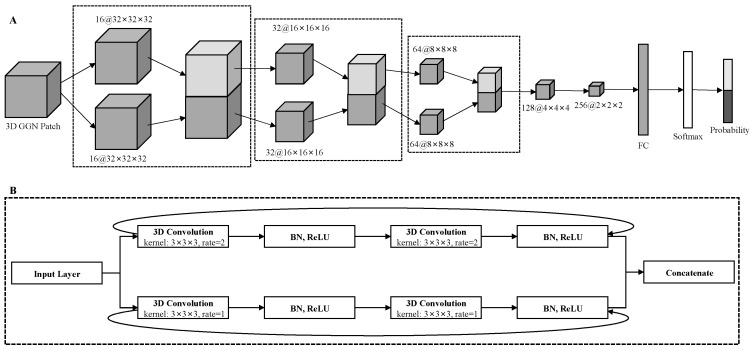
The architecture of the proposed 3D CNN-based DNN model. (**A**) the architecture of 3D ResNet to build the classification models for two tasks. (**B**) the architecture of multi-level concatenated atrous pyramid convolution module showed in the black dotted bordered rectangle of (**A**).

**Figure A3:**
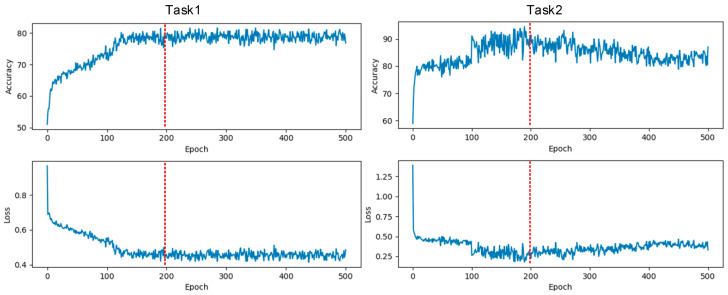
The training accuracy and loss curves for Task1 and Task2, respectively. The red dotted line marks the stopping point in the training process.

Then, a two-stage DNN model was developed by using a sequential convolutional neural network, which was embedded with a residual network (ResNet) and multi-level concatenated atrous pyramid convolution module [29,30]. Appendix B Figure A2 illustrates the architecture of the proposed 3D ResNet-based DNN model. In brief, it consisted of five ResNet blocks and one fully connected layer. In the five ResNet blocks, the former three blocks embedded the atrous convolution structure into the residual block. The details of our proposed DNN model are summarized in Appendix A. Appendix B Figure A3 shows the training accuracy and loss curves for Task1 and Task2, respectively.

The two-stage DNN was implemented using Python 3.7.6 based on the Pytorch 1.5.0 deep learning library, and trained the 3D ResNet on a workstation with 1 NVIDIA GTX 1070 GPU. The source code is open source, at https://github.com/GongJingUSST/GNN_RiskStratification_DNN, 8 November 2020.

### 2.3. MRMC Observer Study Design

An observer study was also conducted to compare the performance of the DNN with six radiologists by testing with validation dataset 2. The six board-certified radiologists from Fudan University Shanghai Cancer Center (Shanghai, China) were enrolled in this MRMC observer study. These radiologists were divided into three groups based on their chest CT imaging interpretation experience, namely, the senior group, middle group, and junior group, respectively. In each group, two radiologists participated in this observer study and each radiologist independently read the CT image without discussion. The senior group enrolled two radiologists who had over 15 years of experience, namely, Reader1 (S.W. with 16 years of experience and 11 years of experience specifically in chest CT interpretation) and Reader2 (H.Z. with 28 years of experience). The middle group enrolled two radiologists who had at least five years of imaging diagnostic experience, namely, Reader3 (H.L. with 11 years of experience) and Reader4 (T.W. with five years of experience). The other two radiologists, namely, Reader5 (T.H. with three years of experience) and Reader6 (M.L. with two years of experience) were enrolled in the junior group. Table 2 listed the reference standard of diagnostics for the two tasks. All readers were aware that all the patients had pathologically confirmed primary lung adenocarcinoma or other benign lesions, but were blinded to the specific histopathological diagnosis and other clinical information to determine the risk of GGNs within five minutes for each case.

### 2.4. Statistical Analysis and Performance Evaluation

The performance of the proposed DNN model was comprehensively evaluated by using eight performance metrics, namely, accuracy, sensitivity, specificity, positive predictive value, negative predictive value, odds ratio, F1 score ($F1=\frac{2\times \mathrm{Precision}\times \mathrm{Recall}}{\mathrm{Precision}+\mathrm{Recall}}$), weighted average F1 score ($F1_{\mathrm{avg}}=\frac{n_{\mathrm{bengign}/\mathrm{non}-\mathrm{IA}}\times F1_{\mathrm{bengign}/\mathrm{non}-\mathrm{IA}}+n_{\mathrm{malignant}/\mathrm{IA}}\times F1_{\mathrm{malignant}/\mathrm{IA}}}{n_{\mathrm{bengign}/\mathrm{non}-\mathrm{IA}}+n_{\mathrm{malignant}/\mathrm{IA}}}$), and Matthews correlation coefficient. The prediction probabilities produced by the DNN model were converted to binary results by using a default decision threshold value of 0.5 as the cut-off.

The area under the receiver operating characteristic (ROC) curve (AUC) and Cohen’s kappa value were also computed to evaluate the model performance. A maximum likelihood-based ROC fitting program (ROCKIT, http://metz-roc.uchicago.edu/MetzROC/software/, 21 October 2013, University of Chicago) was applied to compute AUC values and generate ROC curves. Python programming software was used to compute the performance evaluation metrics and statistical analysis. Several publicly available packages used in this study included SimpleITK, Scikit-learn, SciPy, Matplotlib, NumPy, and Pandas.

## 3. Results

### 3.1. Patient Characteristics

A total 2393 GGNs collected from four centers were enrolled in this study. The characteristics of 2105 patients from four centers are listed in Table 1. Among the four datasets, no statistically significant difference was observed for sex or age (*p* > 0.05). The numbers of GGNs with different pathological type, nodule type, location, and diameter are summarized in Table 1.

### 3.2. DNN Model Validation and Effect of Slice Thickness on Performance

**Figure 2:**
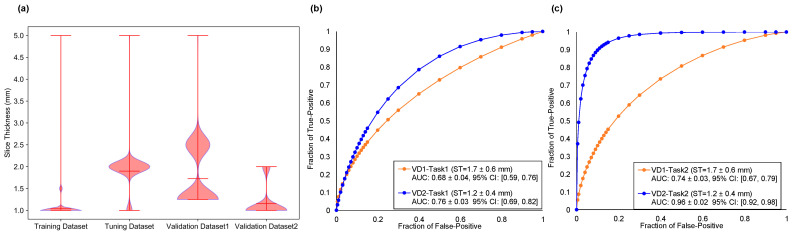
Comparisons of dataset slice thickness and ROC curves for two tasks. (**a**) The violin plots of slice thickness for four datasets. The ST is denoted as slice thickness. (**b**) The ROC curves for Task1 generated by DNN testing on validation dataset (VD) 1 and VD2, respectively. (**c**) The ROC curves for Task2 generated by DNN using testing on VD1 and VD2, respectively.

CT scans collected from four centers had different spatial resolutions, especially in slice thickness. Comparing the pixel spacing of CT slices in the four datasets, no significant differences were observed between them (*p* > 0.05), but the slice thicknesses of CT scans in validation datasets 1 and 2 were significantly different (*p* < 0.05). Figure 2 compares the slice thickness of the four datasets and ROC curves for the two tasks by testing validation datasets 1 and 2, respectively. From the violin plots of slice thickness for the four datasets, it could be seen that the slice thickness of the training dataset and validation dataset 2 was significantly lower than that of the tuning dataset and validation dataset 1 (*p* < 0.00001).

**Figure A4:**
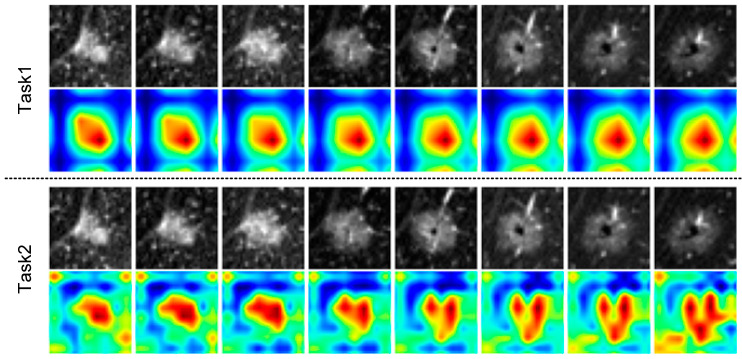
The heat maps of the deep image features for the proposed DNN model.

DNN models trained on validation dataset 2 yielded AUC values of 0.76 ± 0.03 (95% confidence interval (CI) = 0.69–0.82) and 0.96 ± 0.02 (95% CI = 0.92–0.98) for Task1 and Task2, which were significantly higher than models trained on validation dataset 1 of 0.68 ± 0.04 (95% CI = 0.59–0.76) and 0.74 ± 0.03 (95% CI = 0.67–0.79) (*p* < 0.00001). Table 3 lists and summarizes the performance evaluation metrics for two classification tasks by using validation datasets 1 and 2. Appendix B Figure A4 illustrates the heat maps of the deep image features for the proposed DNN model. It can be seen that the DNN model extracted different deep image features for Task1 and Task2. The DNN model mainly focused on the internal regions of GGNs, especially the solid regions.

### 3.3. MRMC Comparison Using an Independent Dataset

**Figure 3:**
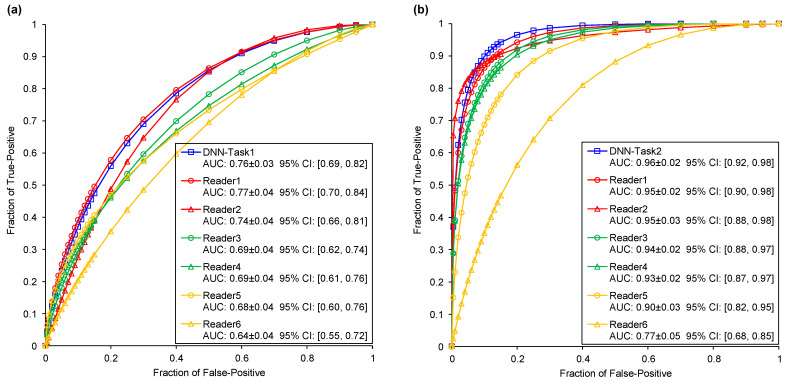
Comparisons of ROC curves and AUC values generated by DNN model and six readers (Table 2). (**a**) The Task1 ROC curves of DNN model and six readers. (**b**) The Task2 ROC curves of DNN model and six readers.

**Figure 4:**
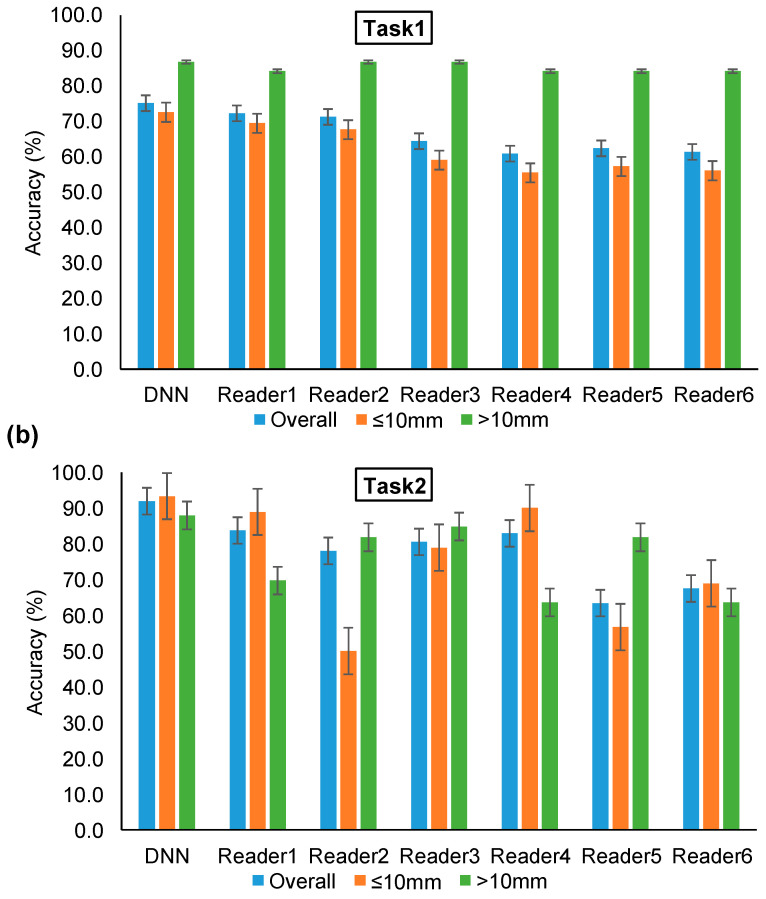
Bar plots of accuracy for DNN model and six readers performing Task1 and Task2, respectively. “Overall” denotes the accuracy of the overall dataset. “≤10 mm” denotes the accuracy for GGNs with a diameter smaller than 10 mm. “>10 mm” denotes the accuracy for GGNs with a diameter larger than 10 mm. (**a**) The accuracy of Task1. (**b**) The accuracy of Task2.

Figure 3 illustrates and compares the two tasks’ ROC curves of the DNN model and six readers by testing on validation dataset 2. In the comparison test of Task1, the senior group, middle group, and junior group yielded average AUC values of 0.76, 0.69, and 0.66, respectively. In the comparison test of Task2, these three radiologist groups obtained average AUC values of 0.95, 0.93, and 0.84, respectively. With an increase in radiologist experience, the AUC values improved accordingly in the two classification tasks. The bar plots of accuracy for the DNN model and six readers in Task1 and Task2 are shown in Figure 4. With an increase in diameter, the benign–malignant classification performance was significantly improved. However, the accuracy of invasiveness prediction changed with the radiologist’s experience. No obvious correlation between GGN diameter and IA/non-IA prediction performance was found in Task2. The confusion matrix of the two tasks generated by the DNN and six readers is shown in Appendix B Table A2. Table 4 compares the performance metrics of the proposed DNN and six readers by testing on validation dataset 2. These comparison indicators revealed that the DNN achieved equivalent or slightly higher performance in comparison with senior radiologists, and a positive trend between GGN risk prediction performance and radiologist’s experience.

### 3.4. Cohen’s Kappa Statistic and Difference Significance Test

**Figure 5:**
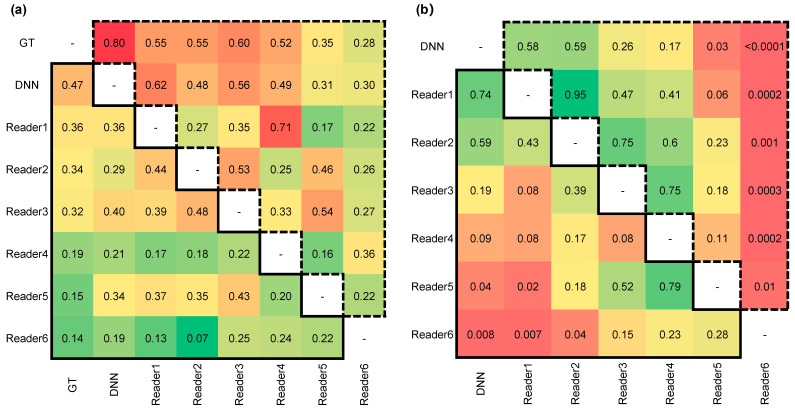
The Cohen’s kappa values and *p*-values for DNN model and six readers. The statistical values contained in a solid line triangle on the bottom left corner represent Task1 testing results. The statistical values contained in a dashed line triangle on top right corner represent Task2 testing results. (**a**) The Cohen’s kappa values for two tasks. (**b**) The *p*-values for two tasks.

Cohen’s kappa values were calculated to measure the inter-rater reliability of the DNN and the six readers compared with the ground truth (GT) of the histopathological results. For the six readers, the binary classification results were generated by categorizing the prediction score of “3” into the high-risk group (i.e., malignant group or IA group). The Cohen’s kappa values and *p*-values for the DNN model and the six readers are presented in Figure 5. Compared with the GT of GGNs, the DNN model and senior group obtained relatively high agreement and consistency decreased with radiologist’s experience. The results suggested no statistically significant difference between the results of the DNN model and senior group (*p* > 0.05).

## 4. Discussion

Management of GGNs is essential for lung adenocarcinoma diagnosis and treatment [31]. Non-invasive CT-based risk stratification of early-stage lung adenocarcinoma provides a potential tool to detect the patients with high-risk malignant and invasive tumors [32]. Unlike the one-stage CT radiomics classification model reported in the literature [10], this study proposed an end-to-end two-stage risk stratification model by using a DNN algorithm, which directly decoded the CT imaging phenotypes of GGNs without manually marked tumor boundaries. The two-stage model not only classified benign and malignant GGNs, but also predicted the invasiveness of malignant tumors by distinguishing IA from non-IA. Thus, our stage-wise risk stratification model provided valuable risk assessment for lung adenocarcinoma, which can help doctors in their management decision making, such as CT follow-up interval, optimal time of biopsy, and appropriate surgical strategy selection.

**Figure A5:**
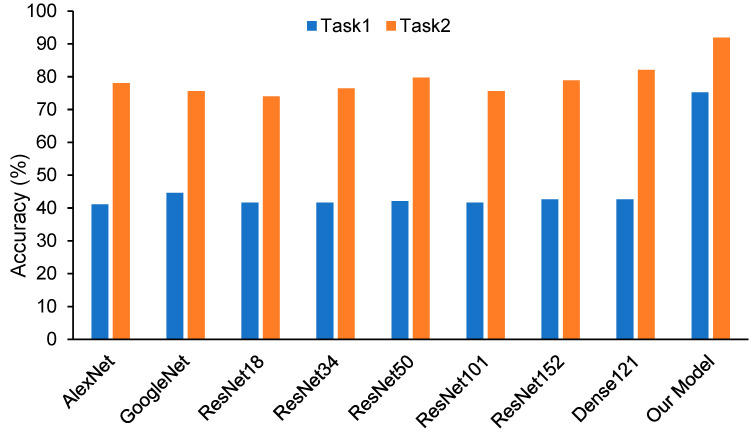
Comparisons of accuracies of the pre-trained models and the proposed model appendix by testing on validation dataset 2. A transfer learning method was used to apply other state-of-the-art pre-trained models to predict the malignancy and invasiveness of GGNs. In the pre-trained model development, the parameters in CNN pooling processes were frozen and the parameters in fully connected or classifier layers were trained. In the model training process, the pre-trained models were the configured with the same parameters as the DNN model.

This multi-center study collected CT scans performed on different scanners in four centers. Thus, the robustness and stability of the proposed model was evaluated on multiple cohorts. Appendix B Figure A5 compares and shows the accuracies of different models by testing on validation dataset 2. Compared with the previous studies [16,18,26,33] and the state-of-the-art pre-trained models, the proposed DNN model showed higher performance in predicting malignancy and invasiveness of GGNs. An MRMC observer study was conducted to further validate and evaluate the performance of the DNN model. The results demonstrated that diagnosis performance had a positive correlation with the experience of the radiologist. The DNN model performed equivalently or even better in comparison with the senior radiologists with over 15 years of experience. Meanwhile, the AUC value of the DNN was significantly higher than the junior radiologists (*p* < 0.05). By evaluating the Cohen’s kappa values, prediction results of the DNN model showed the highest consistency with histopathologically confirmed results. Therefore, the DNN model could provide radiologists a risk indicator for their decision making, which may improve their confidence and accuracy, especially for junior radiologists.

Another finding of this study was that the performance of the DNN model decreased with the increased slice thickness of CT scans. This reflected the fact that the slice thickness of CT scans affected the DNN performance. In both Task1 and Task2, the AUC value of validation dataset 1 (mean slice thickness: 1.73 mm ± 0.64) was lower than that of validation dataset 2 (mean slice thickness: 1.15 mm ± 0.36) (*p* < 0. 00001). Although a few quantitative assessment indicators of validation dataset 1 in Table 3 are a little higher than those of validation dataset 2, the overall evaluation of validation dataset 2’s performance was higher. The performance difference may be because high slice thickness decreased the CT image details. All CT images were resampled to the same voxel spacing, but CT scans with high slice thickness sampled fewer image pixels and provided less information on 3D GGNs for the DNN model. Hence, to improve the model performance, it was necessary to feed and train the DNN with thin-section CT images.

In this study, all the patients enrolled in our dataset underwent thoracic surgery, and the pathologic results of GGNs were obtained from surgically resected specimens. Since regular follow-up examinations were recommended for patients diagnosed with low-risk GGNs in the clinic, most of these surgery patients were diagnosed with high malignancy risk and were suitable for surgery by the chest oncologist. Therefore, the benign GGNs involved in this study were difficult to distinguish from malignant tumors. Compared with the results of Task1, the performance on Task2 generated by the DNN and the six readers were higher. This suggested that using CT scans at one time point to predict the malignancy of GGNs was a more difficult task than classification of IA and non-IA GGNs. In Task1, the accuracy generated by using GGNs with diameter smaller than 10 mm was lower than that for GGNs with diameter larger than 10 mm. It was speculated that this might be due to the increased heterogeneity of the GGN in the CT image as the tumor grows.

Despite the promising results, this study had several limitations. First, although this study collected CT scans from four centers, the comparative radiologist study was still limited to retrospective data from one center. The generalizability and robustness of the proposed DNN model needs to be evaluated by using a more diverse and larger dataset with different institutions, regions, and races. Second, while the two-stage DNN model was developed based on the 3D patch of GGNs (i.e., 32 mm × 32 mm × 32 mm), an entire CT scan and other clinical information (i.e., smoking, medical history, gene information, etc.) [34], which provided additional diagnostic information, has not yet been used to better estimate the risk of GGNs. Follow-up CT scans are also very valuable for GGN diagnosis, but only CT images before surgery were used to develop the DNN model [35]. Aggregating the multi-type diagnosis information of the patient may improve the classification performance further. Third, radiologists read the CT scans under time constraints and blinding to other information in the MRMC study, which was different from a real clinical situation. Thus, insufficient time for the radiologists may have reduced the six readers’ efficiency and performance. Although the performance of the DNN model and radiologists was compared and evaluated in the MRMC study, the correlation and discrepancy were not explored. In a future study, we will continue to explore and investigate the correlations and differences between deep image features and the characteristics defined by radiologists. Lastly, whether using our proposed DNN model can help radiologists improve the diagnosis performance and how to apply it in clinical practice were not investigated in this study.

## 5. Conclusions

In conclusion, a two-stage risk stratification for early lung adenocarcinomas was proposed and developed in this study. Our results revealed (1) a positive trend between the diagnostic performance and radiologist’s experience, (2) the DNN performed equivalently or even better than senior radiologists with over 15 years of experience, and (3) low resolution of CT images decreased the DNN’s performance. The deep learning method illustrated a promising way to realize the risk stratification of GGNs, which may supplement future approaches to GGN diagnosis and support assisted- or second-read workflows.

## Figures and Tables

**Table 1:** Clinical characteristics of the patients in the training, tuning, and validation datasets.

Characteristic	Training Dataset (*n* = 1476)	Tuning Dataset (*n* = 431)	Validation Dataset 1 (*n* = 284)	Validation Dataset 2 (*n* = 202)
Mean Age, y (SD)	53.8 (±11.0)	54.3 (±11.8)	57.9 (±11.1)	54.7 (±10.7)
Sex, No. (%)				
Male	409 (31.4)	129 (35.3)	103 (39.2)	57 (32.6)
Female	893 (68.6)	236 (64.7)	160 (60.8)	118 (67.4)
WHO pathological type, No. (%)				
Benign/AAH	206 (13.9)	73 (16.9)	38 (13.4)	79 (39.1)
AIS	623 (42.2)	77 (17.9)	55 (19.4)	53 (26.2)
MIA	261 (17.7)	8 (1.9)	64 (22.5)	33 (16.3)
IA	386 (26.2)	273 (63.3)	127 (44.7)	37 (18.3)
Location, No. (%)				
RUL	543 (36.8)	157 (36.4)	118 (41.5)	80 (39.6)
RML	110 (7.5)	31 (7.2)	17 (6.0)	14 (6.9)
RLL	270 (18.3)	76 (17.6)	48 (16.9)	27 (13.4)
LUL	384 (26.0)	109 (25.3)	71 (25.0)	53 (26.2)
LLL	169 (11.4)	58 (13.5)	30 (10.6)	28 (13.9)
Nodule type on CT scan, No. (%)				
pGGN	1093 (74.1)	308 (71.5)	102 (35.9)	175 (86.6)
mGGN	383 (25.9)	123 (28.5)	182 (64.1)	27 (13.4)
Diameter (mm), No. (%)				
(3,10]	888 (60.2)	258 (59.9)	135 (47.5)	164 (81.2)
(10,20]	452 (30.6)	143 (33.2)	120 (42.3)	36 (17.8)
(20,30]	136 (9.2)	30 (6.9)	29 (10.2)	2 (1.0)

Abbreviations and definitions: WHO = World Health Organization; AAH = atypical adenomatous hyperplasia; AIS = adenocarcinoma in situ; MIA = minimally invasive adenocarcinoma; RUL = right upper lobe; RML = right middle lobe; RLL = right lower lobe; LUL = left upper lobe; LLL = left lower lobe; pGGN = pure ground glass opacity nodule; mGGN = mixed ground glass nodule.

**Table 2:** The reference standards of imaging diagnosis for two tasks. To score the malignancy risk and invasive risk of each GGN, two diagnostic reference standards were developed by grading the risk with five grades.

Task1		Task2	
Reference Standard of Diagnosis	Score	Reference Standard of Diagnosis	Score
Highly suspicious normal/benign	1	Highly unlikely IA	1
Moderately suspicious benign	2	Moderately unlikely IA	2
Indeterminate/probably benign	3	Indeterminate	3
Moderately suspicious malignant	4	Moderately suspicious IA	4
Highly suspicious malignant	5	Highly suspicious IA	5

**Table 3:** Performance evaluation metrics for two classification tasks by using validation datasets 1 and 2, respectively.

Evaluation Metric	Task1		Task2	
	VD1	VD2	VD1	VD2
Accuracy (%)	81.6	75.2	63.8	91.9
Sensitivity (%)	90.7	83.7	86.6	81.1
Specificity (%)	21.6	62	39.5	96.5
PPV (%)	88.5	77.4	60.4	90.9
NPV (%)	25.8	71	73.4	92.2
OR	2.7	8.4	4.2	118.6
F1 (%)	89.6	80.5	71.2	85.7
F1avg (%)	80.9	74.9	61.6	91.7
MCC (%)	13.2	47.1	29.7	80.3

Abbreviations and definitions: VD = validation dataset; PPV = positive predictive value; NPV = negative predictive value; OR = odds ratio; MCC = Matthews correlation coefficient.

**Table 4:** Performance comparisons of the proposed DNN and six readers by testing on validation dataset 2.

Evaluation Index	Task1							Task2						
	DNN	R1	R2	R3	R4	R5	R6	DNN	R1	R2	R3	R4	R5	R6
Accuracy (%)	75.2	69.8	71.3	64.4	60.9	62.4	61.4	91.9	83.7	78.0	80.5	82.9	63.4	67.5
Sensitivity (%)	83.7	83.7	92.7	94.3	52.0	85.4	76.4	81.1	48.6	91.9	94.6	45.9	100.0	59.5
Specificity (%)	62.0	54.4	38.0	17.7	74.7	26.6	38.0	96.5	98.8	72.1	74.4	98.8	47.7	70.9
PPV (%)	77.4	74.1	69.9	64.1	76.2	64.4	65.7	90.9	94.7	58.6	61.4	94.4	45.1	46.8
NPV (%)	71.0	68.3	76.9	66.7	50.0	53.8	50.8	92.2	81.7	95.4	97.0	81.0	100.0	80.3
F1 (%)	80.5	78.6	79.7	76.3	61.8	73.4	70.7	85.7	64.3	71.6	74.5	61.8	62.2	52.4
F1avg (%)	74.9	71.6	68.4	57.4	61.1	58.6	60.0	91.7	81.9	78.9	81.3	80.8	63.9	68.4
MCC (%)	47.1	40.2	37.9	19.2	26.5	14.8	15.5	80.3	60.3	58.8	63.5	58.1	46.4	28.7

Abbreviations and definitions: PPV = positive predictive value; NPV = negative predictive value; MCC = Matthews correlation coefficient; R = reader.

## Data Availability

The source code is open source at https://github.com/GongJingUSST/GNN_RiskStratification_DNN (accessed on 8 November 2020).

## Appendix A

The detailed description of the DNN model.

In order to develop a DNN model, a number of layers, including convolutional layers, max-pooling layers, and fully connected (FC) layers, were used to build a sequential CNN. With the number of layers increasing, the DNN encountered the problem of the vanishing/exploding gradient. To address this issue, a residual network (ResNet) introduced a technique called “skip connection” by skipping training from a few layers and connecting directly to the output. The architecture of ResNet was an effective tool to improve the DNN’s performance and is applied in many medical image classification or segmentation fields. In this study, a 3D ResNet was used to develop the DNN model for two-stage risk stratification of GGNs.

To expand the reception field without increasing the number of parameters and dimensional size, a 3D atrous convolution was used to build the ResNet block. Then, the proposed DNN obtained a multi-scale of feature maps and expanded the depth of layers by combing atrous convolution with ResNet architecture. The two-stage DNN used A) the architecture of 3D ResNet to build the classification models for two tasks, respectively.

In brief, it consisted of five ResNet blocks and one FC layer. In the five ResNet blocks, the former three blocks embedded the atrous convolution structure into the residual block, and were called B) the multi-level concatenated atrous pyramid convolution (MLAPC) module. In each MLAPC block, two standard convolution layers with a 3 × 3 × 3 convolution kernel and a dilation rate of r = 1 and two atrous convolution layers with a 3 × 3 × 3 convolution kernel and a dilation rate of r = 2 were used to obtain feature maps of different receptive fields. The batch normalization (BN) layer and the rectified linear unit (ReLU) were placed after each convolution layer in sequence. At the bottom of the MLAPC block, these multi-scale image features extracted by the MLAPC module were concatenated before sending to the next module. The max-pooling layer was connected with each ResNet block to reduce the dimension of features. The other two ResNet blocks consisted of two standard convolution layers with a 3 × 3 × 3 convolution kernel and a dilation rate of r = 1, two BN layers, and two ReLUs. The FC layer and a softmax activation function were used to build the classification head to output the risk probability of each task.

The proposed DNN model performed two classification tasks of the GGN risk stratification process, and used cross-entropy to define the loss function for each task. An adaptive moment estimation (Adam) optimizer was used to minimize the cross-entropy loss between the model outputs and target classification labels. To train the two-stage DNN, Adam optimizers were configured with a learning rate of 0.001 and weight decay of 1.0 × 10^−4^ for two tasks. In the optimization process, the learning rates of the parameter group were decayed after 200 and 100 epochs by gamma values of 0.1 and 0.5 for the two-stage DNNs. For each stage of DNN model training, the training dataset was resampled with a sample number ratio of 1:1 for the two classes by using the data augmentation techniques. In the training process, a batch size of 64 was set to update the parameters. To improve the robustness of the DNN, a mix-up data augmentation technique with an α value of 0.5 was applied to train the models. We stopped the training early after 200 epochs. No dropout operation was used in the network.

## Appendix B

**Table A1:** The statistics of the manufacturer and convolutional kernel for CT images in our datasets.

Dataset	Manufacturer	Manufacturer Model Name	Convolutional Kernel	Number
Training Dataset (NCT = 1302)	Philips	Brilliance 64	B	59
			L	6
	SIEMENS	SOMATOM Definition AS	B31f	146
			B70f	1
		SOMATOM Definition AS	B31f	135
			B75f	1
		Sensation 40	B31f	1
		Sensation 64	B30f	174
			B31f	718
			B50f	1
			B70f	59
	TOSHIBA	Aquilion ONE	FC08	1
Tuning Dataset (NCT = 365)	Philips	Brilliance 16	B	2
			L	293
	TOSHIBA	Aquilion	FC51	1
			FC52	24
		Aquilion ONE	FC51	34
			FC52	8
			FC86	2
	United Imaging Healthcare	uCT 528	B_SHARP_C	1
Validation Dataset 1 (NCT = 263)	GE MEDICAL SYSTEMS	LightSpeed VCT	BONEPLUS	17
			CHST	88
			STANDARD	2
		LightSpeed16	BONEPLUS	28
			LUNG	33
			STANDARD	44
		Optima CT540	BONEPLUS	37
			LUNG	14
Validation Dataset2 (NCT = 175)	Philips	Brilliance 40	C	11
		Ingenuity Flex	C	1
			YA	1
		iCT 256	B	38
			L	3
	SIEMENS	SOMATOM Definition AS+	B31f	106
	United Imaging Healthcare	uCT 510	B_SOFT_C	11
		uCT 760	B_SHARP_AB	4

**Table A2:** The confusion matrix of two tasks generated by DNN and six readers.

Model	Task1			Task2		
	Ground Truth	Predicted Benign	Predicted Malignant	Ground Truth	Predicted Non-IA	Predicted IA
DNN	Benign	49	30	Non-IA	83	3
	Malignant	20	103	IA	7	30
Reader1	Benign	43	36	Non-IA	85	1
	Malignant	20	103	IA	19	18
Reader2	Benign	30	49	Non-IA	62	24
	Malignant	9	114	IA	3	34
Reader3	Benign	14	65	Non-IA	64	22
	Malignant	7	116	IA	2	35
Reader4	Benign	59	20	Non-IA	85	1
	Malignant	59	64	IA	20	17
Reader5	Benign	21	58	Non-IA	41	45
	Malignant	18	105	IA	0	37
Reader6	Benign	30	49	Non-IA	61	25
	Malignant	29	94	IA	15	22
